# Contribution to the DNA barcode reference library for Réunion Pteridophyta

**DOI:** 10.3897/BDJ.14.e199400

**Published:** 2026-09-01

**Authors:** Mirana Gauche, Némo Fontanié, Edmond Grangaud, Nicholas Wilding, Rodolphe Rougerie, Marie Lapègue, Claudine Ah-Peng

**Affiliations:** 1 UMR PVBMT, Université de la Réunion, Saint-Pierre, Réunion UMR PVBMT, Université de la Réunion Saint-Pierre Réunion; 2 Ecology and genetics unit, University of Oulu, Oulu, Finland Ecology and genetics unit, University of Oulu Oulu Finland; 3 Les Cytises, Saint-Pierre, Réunion Les Cytises Saint-Pierre Réunion; 4 Missouri Botanical Garden, Missouri, United States of America Missouri Botanical Garden Missouri United States of America; 5 Muséum national d'Histoire Naturelle, Paris, France Muséum national d'Histoire Naturelle Paris France; 6 Observatoire des Sciences de l'Univers de La Réunion (OSU-Réunion), Université de la Réunion, CNRS, IRD, Météo France, Saint-Denis, Réunion Observatoire des Sciences de l'Univers de La Réunion (OSU-Réunion), Université de la Réunion, CNRS, IRD, Météo France Saint-Denis Réunion

**Keywords:** DNA barcode, introduced species, pteridophytes, reference database, Réunion Island, trnH–psbA

## Abstract

**Background:**

Expanding DNA barcode reference libraries is essential to support ecological surveys, conservation assessments and future metabarcoding applications. Réunion Island (Mascarene's Archipelago) hosts a rich diversity of pteridophytes despite its small surface area. Although the chloroplastic rbcL sequences are available for several fern taxa in public databases, additional regions with high variability such as trnH–psbA are still under-represented for the Réunion pteridoflora. This dataset has been produced in the framework of the FRBOL (FRench Barcode Of Life) network.

**New information:**

The current curated DNA barcode dataset contains 84 pteridophyte species from Réunion Island, representing approximately 25% of the pteridophyte species present on the Island. All specimens were morphologically identified by a specialist and are preserved as vouchers in the public herbarium of the University of Réunion (REU). A total of 163 sequences of rbcLa (86) and trnH–psbA (77) were generated and deposited in the Barcode of Life Data System (BOLD) with complete associated metadata and photos. While numerous rbcLa sequences were already available on GenBank, our dataset provides newlygenerated sequences linked to curated vouchers on BOLD and substantially increases the representation of trnH–psbA for the Island’s pteridophytes. Additionally, this work documents the first record of *Pteris ensiformis* Burm.f., an exotic species for Réunion. Morphological examination allowed the identification of this newly-recorded species, which was corroborated by molecular analyses.

## Introduction

DNA barcoding is a standardised molecular approach that uses short, taxonomically informative DNA regions to enable rapid and reliable species identification (Hebert et al. 2003). In plants, the combination of the two plastid loci rbcL and matK was earlier proposed as barcode markers (Group CPW et al. 2009) and have since been widely adopted in floristic surveys, taxonomic studies and biodiversity assessments. Over the past decades, advances in high-throughput sequencing approaches have significantly improved the accuracy, sensitivity and speed of DNA barcoding (Antil et al. 2022). Accordingly, biodiversity monitoring programmes increasingly incorporate molecular metabarcoding approaches. However, the reliability of these applications depends on high-quality, voucher-linked reference libraries, as inaccuracies in public sequence data can compromise species identification and subsequent ecological interpretations (Collins and Cruickshank 2012). The development of comprehensive, taxonomically validated DNA barcode reference databases, therefore, remains a central priority for improving species identification accuracy and ensuring reproducibility in molecular biodiversity studies.

The French Barcode of Life network (FRBOL), led by the National Museum of Natural History (MNHN) with support from the French Biodiversity Agency (OFB), aims to coordinate and strengthen activities related to the use of DNA barcodes at the national level, in particular to provide access to validated and reliable reference sequences for the molecular taxonomic identification of taxa present in France. In 2024, a cross-referencing of the data available on the international Barcode of Life Datasystems (BOLD) platform with the French national taxonomic reference database (TaxRef) revealed obvious gaps in the sequence data for mainland France and its overseas territories. As part of FRBOL, the PVBMT research unit (https://umr-pvbmt.cirad.fr/) is collaborating with the MNHN to produce DNA barcode sequences for arthropods, flora and fungi of Réunion.

Pteridophytes are often highly represented in oceanic island floras compared to continental regions, likely due to their effective spore-mediated dispersal and establishment capacity (Tryon 1970,Smith 1972,Kreft et al. 2010). The national reference database TaxRef v.18 (Gargominy et al. 2025) lists 320 valid pteridophyte species names for Réunion, including native and introduced species. All status categories (native, endemic and introduced) were considered in this dataset, as any of these taxa may be detected in metabarcoding or environmental DNA surveys and, therefore, require reliable reference sequences for accurate taxonomic assignment. Although rbcL is widely used for plant barcoding due to its high amplification success and alignment reliability, it may lack sufficient variability to discriminate closely-related species. For example, in genera, such as *Asplenium*, *Pteris* and some members of Dryopteridaceae, rbcL alone provides insufficient resolution amongst closely-related species, necessitating the use of additional markers (Ebihara et al. 2010,Caetano Wyler and Naciri 2016, Trujillo-Argueta et al. 2021). Since there are no universal primers to amplify matK for pteridophytes (Trujillo-Argueta et al. 2021), this marker was not considered here. Large-scale floristic studies have demonstrated the effectiveness of the trnH-psbA locus in species descrimination (Caetano Wyler and Naciri 2016) and this marker has likewise shown strong discriminatory power in pteridophytes (Ebihara et al. 2010, Ma et al. 2010, Nitta et al. 2017, Trujillo-Argueta et al. 2021). Accordingly, the plastid markers rbcL and trnH–psbA were generated in this study, resulting in the first BOLD-compliant reference dataset for the flora of Réunion, including complete metadata, GPS coordinates, trace files and specimen images.

## General description

### Purpose

This dataset represents a first contribution towards the creation of a comprehensive DNA barcode reference library for the flora of Réunion Island, focusing on pteridophyte species. Such a reference library is expected to facilitate DNA-based species identification in both conventional molecular studies and DNA metabarcoding surveys, while providing a valuable resource for taxonomic and biogeographic research.

## Project description

### Title

Contribution to the DNA barcode reference library of Réunion Island pteridoflora.

### Personnel

Rodolphe Rougerie (project coordinator of FRBOL at MNHN), Claudine Ah-Peng (project coordinator at REU herbarium), Edmond Grangaud (specialist of pteridophytes of the Mascarenes: identification of the specimens), Némo Fontanie (student: field collection and laboratory work), Nicholas Wilding (plant systematics), Mirana Gauche (REU herbarium manager: specimens curation and data management).

### Study area description

**Figure 1.**
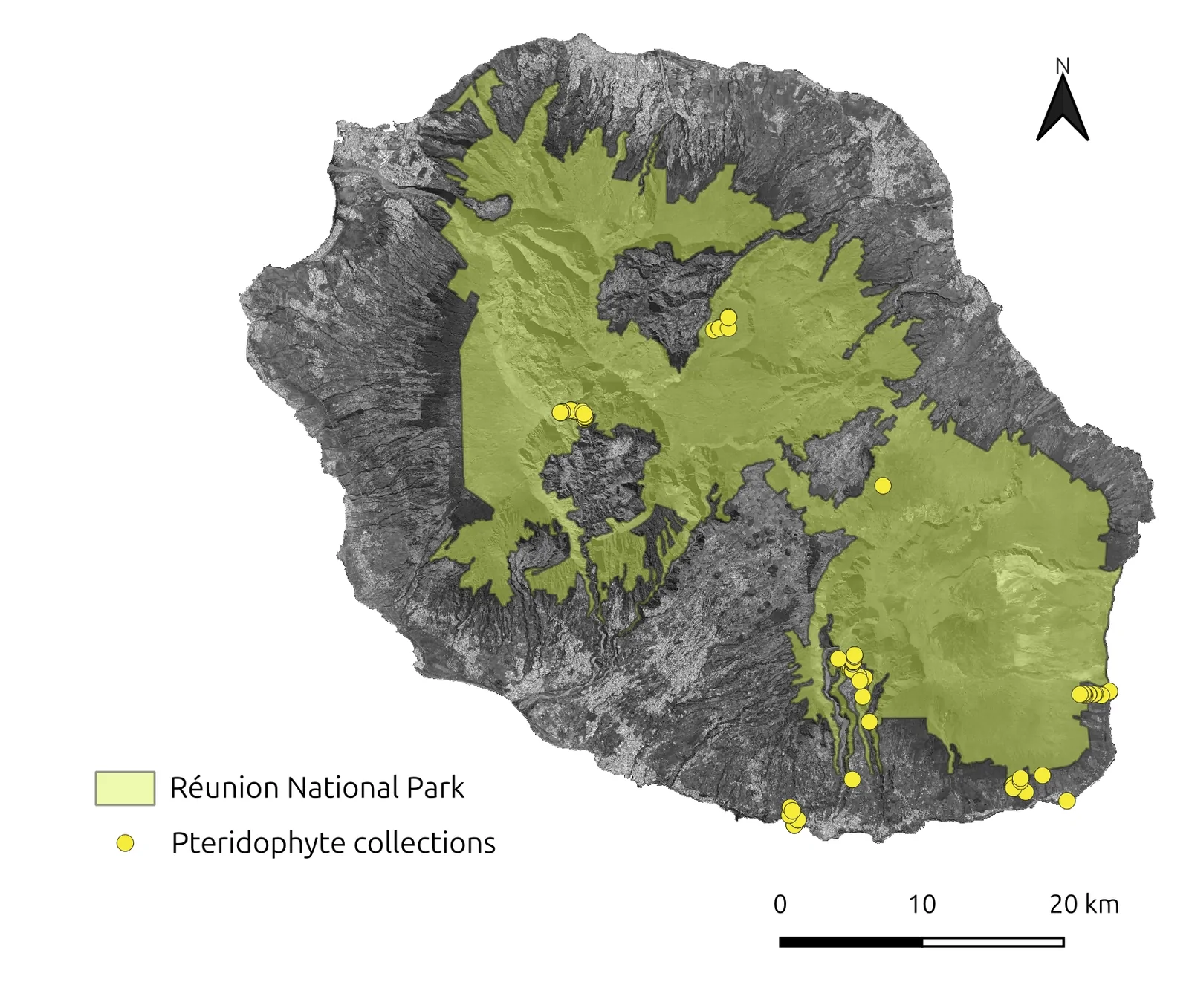
Map of the localities where the samples in the dataset were collected in Réunion Island.

The samples were collected at different localities in natural environnements in Réunion Island (Fig. 1). Réunion is a French overseas Department located in the south-western Indian Ocean. It is a volcanic island with an area of 2,512 km^2^ that is part of the Mascarene's Archipelago. Samples included in this dataset were collected in the municipalities of Cilaos, Plaine-des-Palmistes, Saint-Benoît, Saint-Joseph and Saint-Philippe, representing different habitats (Table 1).

### Design description

Samples were collected in the field, dried and mounted as herbarium specimens. These were morphologically identified by a collector and verified by an expert, photographed and databased. Herbarium specimens are stored at REU Herbarium (Université de La Réunion 2021) and digitised specimens are accessible on the herbarium's website (https://collections-umr-pvbmt.cirad.fr). Small pieces of leaves were dried in silica gel and used for DNA barcoding. DNA extraction and sequencing were performed at the Canadian Centre for DNA Barcoding (CCDB) and results were made available on BOLD. Depositing the dataset in BOLD guarantees its long-term preservation, standardisation and interoperability of barcode records. The system requires structured and voucher-linked metadata, including georeferenced sampling details and sequence quality controls, thereby strengthening data traceability, reliability and reproducibility. Furthermore, BOLD provides integrated analytical tools for sequence comparison, BIN clustering and taxonomic assessment, supporting applications in species identification and metabarcoding. Making the dataset publicly available through this platform also enhances transparency, facilitates data reuse and fosters integration into wider biodiversity research frameworks.

### Funding

The project BIOSCAN-France – supported by the French Biodiversity Agency (OFB) and led by the Museum of Natural History (PI: Antoine Lévêque) is acknowledged for the funding of sequencing of the specimens and the project BioMonI – Biodiversity monitoring of island ecosystems project under the 2022-2023 BiodivMon joint call (PI: C. Ah-Peng) for the collection and curation of specimens. Némo Fontanie benefitted from a learning internship agreement with Erasmus+ for his stay at the UMR PVBMT, University of Réunion.

## Sampling methods

### Study extent

Réunion Island (Mascarene Archipelago, Indian Ocean)

### Sampling description

This study covers pteridophyte species present on Réunion Island (Mascarene's Archipelago, Indian Ocean). Sampling was conducted in September and October of 2024 and includes 84 species, most of which are native to the Island. Specimens were collected in different habitats at 10 different localities (Table 1). A representative part of the plant was mounted as a herbarium specimen and fragments of leaf tissue were sent for sequencing.

### Quality control

Morphological identification of the plants was carried out by E. Grangaud, a specialist of Mascarene pteridophytes. Taxon names were checked against the online database 'World Ferns' (Hassler 1994) which is the reference database used by the international pteridological community. Data associated with the herbarium samples are managed in a curated database. Each sequence was manually verified in GenBank and BOLD to confirm that there were no inconsistencies arising from potential misidentification, contamination or low-quality sequences.

### Step description

Specimens were collected in September and October 2024 in different habitats at 10 different localities in Réunion Island. Representative parts of the specimens were collected, pressed and dried to make vouchers. Fragments of leaf tissue were placed in silica gel for molecular analysis.

The following steps were carried out on the Plant Protection Platform (3P, IBISA). Morphological identifications were carried out under a binocular loupe by an expert (E. Grangraud), based on existing literature (Bosser et al. 2008, Grangaud 2010). The samples were mounted on herbarium sheets and metadata labels were generated. The herbarium sheets were photographed using a camera mounted on a reproduction stand. The samples were added to the REU Herbarium database and are available online (https://collections-umr-pvbmt.cirad.fr/). The leaf tissue samples were prepared according to the CCDB sampling instructions. Tissue pieces ca. 1 × 0.5 cm in area were subsampled with clean forceps and placed in a sampling tube. Metadata and photos were also imported into BOLD before sending the sampling tubes for sequencing.

The sampling tubes were sent to CCDB for DNA extraction and sequencing according to their protocols. Trace files and clean sequences of rbcLa and trnH-psbA were made available on BOLD. Each sequence was individually compared against GenBank and BOLD databases to ensure that there were no discrepancies due to misidentification, contamination or poor sequence quality. One specimen with contaminated sequences was removed from the dataset.

## Geographic coverage

### Description

This study was conducted in different localities in Réunion Island (Mascarene's Archipelago), France (Fig. 1).

### Coordinates

-21.378 and -21.054 Latitude; 55.805 and 55.434 Longitude.

## Taxonomic coverage

### Description

**Figure 2.**
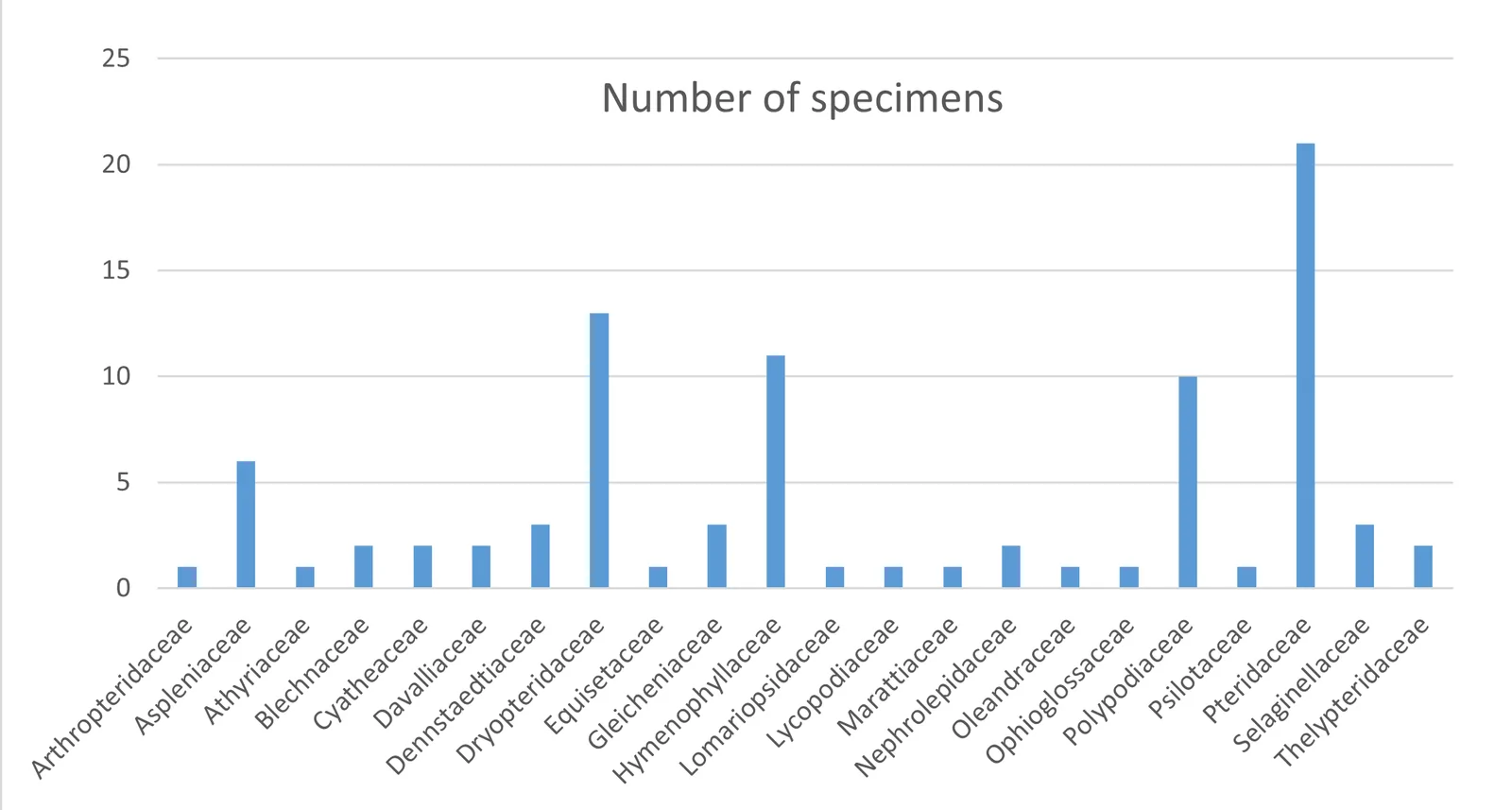
Number of specimens per family present in the dataset.

**Figure 3.**
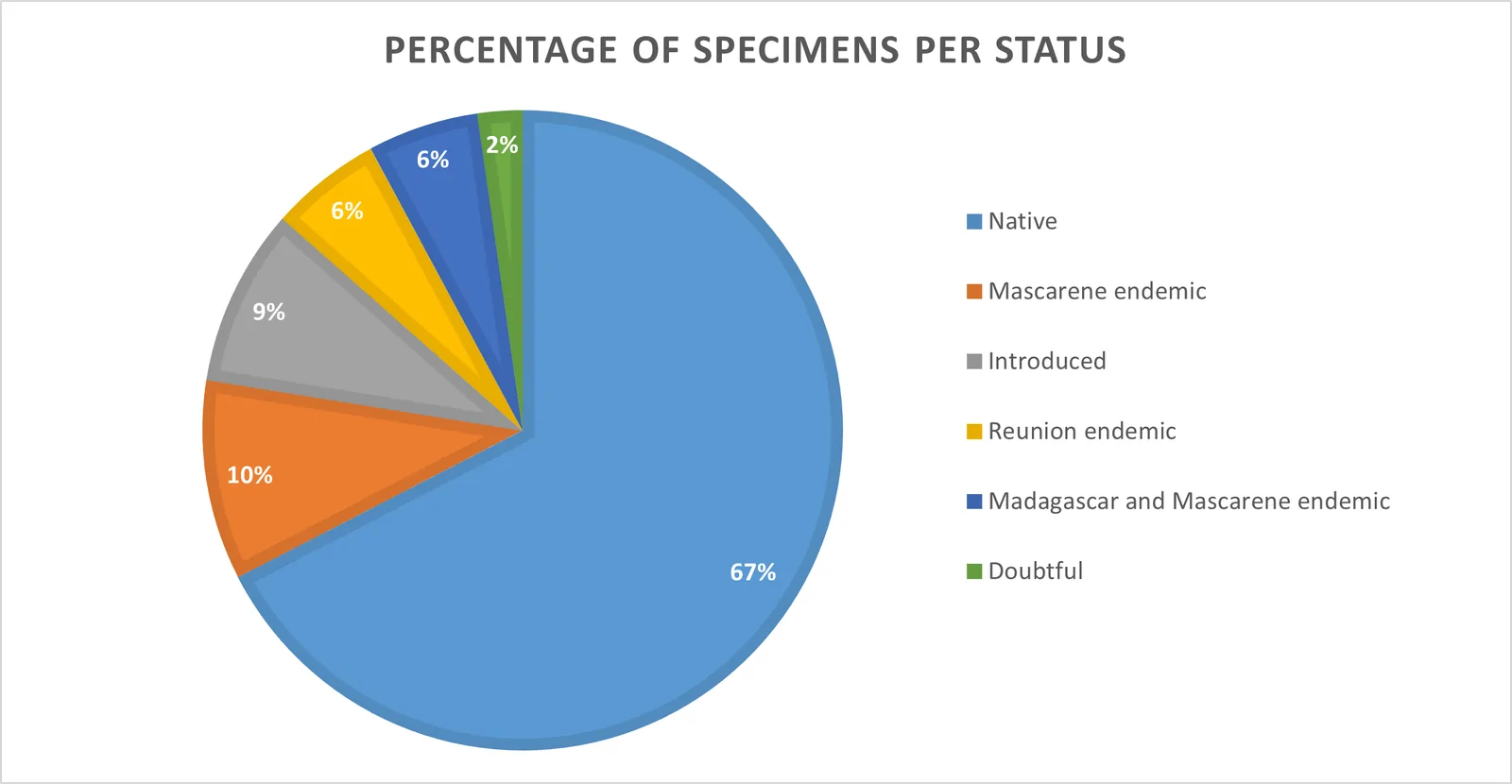
Status for Réunion according to TaxRef v.18 and the *Index de la flore vasculaire (Trachéophytes) de la Réunion*: Native, Mascarene endemic, Introduced, Réunion endemic, Madagascar and Mascarene endemic or Doubtful.

This dataset contains 89 specimens morphologically identified to species level. The dataset covers 84 species belonging to 22 families (Fig. 2.) The most represented families are Pteridaceae (24%), Dryopteridaceae (15%) and Hymenophyllaceae (12%). The status of each species was checked against TaxRef v.18.0 (Gargominy et al. 2025) and the *Index de la flore vasculaire (Trachéophytes) de la Réunion* (Boullet 2020); specimens in this dataset were mostly (67%) native to Réunion (Fig. 3).

### Taxa included

**Table:** 

Rank	Scientific Name	
species	*Abrodictyum parviflorum* (Poir.) Bauret et Dubuisson	
species	*Acrostichum aureum* L.	
species	*Adiantum hispidulum* Sw.	
species	*Adiantum poiretii* Wikstr.	
species	*Adiantum raddianum* C. Presl	
species	*Adiantum reniforme* L.	
species	*Adiantum trapeziforme* L.	
species	*Aleuritopteris farinosa* (Forssk.) Fée	
species	*Alsophila borbonica* (Desv.) R.M. Tryon	
species	*Amauropelta heteroptera* (Desv.) Holttum	
species	*Anthonya hirta* (Sw.) Windham & Pryer	
species	*Antrophyopsis boryana* (Willd.) Schuettp.	
species	*Arthropteris orientalis* (J.F. Gmel.) Posth.	
species	*Asplenium adiantum-nigrum* L.	
species	*Asplenium aethiopicum* (Burm. f.) Bech.	
species	*Asplenium boltonii* Hook. ex Brause et Hieron.	
species	*Asplenium monanthes* L.	
species	Asplenium nitens Sw.	
species	*Asplenium pellucidum* Lam.	
species	*Blotiella pubescens* (Willd. ex Kaulf.) R.M. Tryon	
species	*Ceradenia leucosora* (Bojer ex Hook.) Parris	
species	*Cochlidium serrulatum* (Sw.) L.E. Bishop	
species	*Crepidomanes bipunctatum* (Poir.) Copel.	
species	*Ctenitis borbonica* (Baker) Tardieu	
species	*Ctenitis humida* (Cordem.) Holttum	
species	*Ctenitis maritima* (Cordem.) Tardieu	
species	*Davallia denticulata* (Burm. f.) Mett. ex Kuhn	
species	*Davallia repens* (L. f.) Kuhn	
species	*Dicranopteris linearis* (Burm. f.) Underw.	
species	*Didymoglossum cuspidatum* (Willd.) Ebihara et Dubuisson	
species	*Diplazium proliferum* (Lam.) Thouars	
species	*Dryopteris aquilinoides* (Desv.) C. Chr.	
species	*Dryopteris bernieri* Tardieu	
species	*Elaphoglossum acrostichoides* (Hook. et Grev.) Schelpe	
species	*Elaphoglossum hybridum* (Bory) Brack.	
species	*Elaphoglossum lancifolium* (Desv.) C.V. Morton	
species	*Elaphoglossum lepervanchii* (Bory ex Fée) T. Moore	
species	*Elaphoglossum macropodium* (Fée) T. Moore	
species	*Elaphoglossum spatulatum* (Bory) T. Moore	
species	*Elaphoglossum splendens* (Bory ex Willd.) Brack.	
species	*Equisetum ramosissimum* Desf.	
species	*Haplopteris ensiformis* (Sw.) E.H. Crane	
species	*Haplopteris graminea* (Poir.) C.W.Chen, S.Linds. & Schuettp.	
species	*Histiopteris incisa* (Thunb.) J. Sm.	
species	*Hymenophyllum digitatum* (Sw.) Fosberg	
species	*Hymenophyllum hygrometricum* (Poir.) Desv.	
species	*Hymenophyllum inaequale* (Poir.) Desv.	
species	*Hymenophyllum sibthorpioides* (Bory ex Willd.) Mett. ex Kuhn	
species	*Lepisorus excavatus* (Bory ex Willd.) Ching	
species	*Lepisorus spicatus* (L. f.) Li Wang	
species	*Lomaridium attenuatum* (Sw.) Gasper & V.A.O. Dittrich	
species	*Lomariopsis pollicina* (Willemet) Mett. ex Kuhn	
species	*Loxogramme lanceolata* (Sw.) C. Presl	
species	*Microsorum scolopendria* (Burm.f.) Copel.	
species	*Nanogrammitis obtusa* (Willd. ex Kaulf.) Parris, Li Bing Zhang, XMZhou & Liang Zhang	
species	*Nephrolepis biserrata* (Sw.) Schott	
species	*Nephrolepis cordifolia* (L.) C. Presl	
species	*Oleandra distenta* Kunze	
species	*Ophioderma pendulum* (L.) C. Presl	
species	*Palhinhaea cernua* (L.) Franco et Vasc.	
species	*Parablechnum marginatum* (Fée) Gasper & Salino	
species	*Pellaea viridis* (Forssk.) Prantl	
species	*Pellaeopsis dura (J.Sm.) Windham*	
species	*Phlebodium aureum* (L.) J Sm.	
species	*Pityrogramma calomelanos* (L.) Link	
species	*Polyphlebium diaphanum* (Kunth) Ebihara & Dubuisson	
species	*Psilotum nudum* (L.) P. Beauv.	
species	*Pteridium aquilinum* (L.) Kuhn	
species	*Pteris ensiformis* Burm. f.	
species	*Pteris pallens* (Sw.) Mett.	
species	*Pteris scabra* Bory ex Willd.	
species	*Pteris vittata* L.	
species	*Ptisana fraxinea* (Sm.) Murdock	
species	*Pyrrosia lanceolata* (L.) Farw.	
species	*Rouhania parvula* (Bory ex Willd.) Li Bing Zhang, XMZhou, Jian Jun Yang & Liang Zhang	
species	*Rouxopteris boryi* (Kunze) H.M. Liu	
species	*Selaginella distachya* Cordem.	
species	*Selaginella sinuosa* (Desv.) Alston	
species	*Selaginella viridula* (Bory ex Willd.) Spring	
species	*Sphaeropteris cooperi* (Hook. ex F. Muell.) R.M. Tryon	
species	*Sphaerostephanos elatus* (Bojer) Holttum	
species	*Sticherus flagellaris* (Bory ex Willd.) Ching	
species	*Vandenboschia gigantea* (Bory ex Willd.) Pic.Serm.	
species	*Vittaria isoetifolia* Bory	

## Temporal coverage

### Notes

Samples were collected between September and November 2024.

## Collection data

### Collection name

Herbier Universitaire de La Réunion. Accessible at: https://collections-umr-pvbmt.cirad.fr/

### Collection identifier

REU

### Specimen preservation method

dry

## Usage licence

### Usage licence

Creative Commons Public Domain Waiver (CC-Zero)

## Data resources

### Data package title

Pteridophytes of Réunion Island barcoded in the context of FRBOL project

### Resource link

http://www.boldsystems.org/index.php/Public_SearchTerms?query=DS-PTEREU

### Number of data sets

1

### Data set 1.

#### Data set name

DS-PTEREU

#### Data format

json, tsv, dwc, fasta

#### Download URL

https://doi.org/10.5883/DS-PTEREU

#### Description

The dataset entitled *“*Pteridophytes of Réunion Island barcoded in the context of the FRBOL project” is available from the BOLD Public Data Portal in DwC, JSON and TSV formats, with sequences downloadable as FASTA files. Records can also be queried directly within BOLD. The column names listed below correspond to those that are relevant for this dataset in the TSV export format. Field definitions follow the Barcode Community Data Model (BCDM) as provided by BOLD Systems (GitHub repository, accessed in February 2026). Note that the mapping of BCDM to BOLD and BCDM to DwC are also available on the repository.

**Data set 1.** 

Column label	Column description
processid	BOLD System generated unique identifier for the sample being sequenced.
sampleid	User generated identifier for the sample being sequenced, often identical to the Field ID or Museum ID.
fieldid	Specimen or sample identifier generated in the field or lot number for a bulk collection event.
museumid	Catalogue number from a museum collection.
record_id	A BOLD generated identifier for the marker and specimen sequence combination.
inst	Institution storing the physical specimen.
kingdom	Taxonomy: Kingdom.
phylum	Taxonomy: Phylum.
class	Taxonomy: Class.
order	Taxonomy: Order.
family	Taxonomy: Family.
subfamily	Taxonomy: Subfamily.
tribe	Taxonomy: Tribe
genus	Taxonomy: Genus.
species	Taxonomy: Species.
identification	Taxonomic identification of the specimen.
identification_method	The method used to identify the specimen.
identification_rank	Taxonomic rank of the identification associated with the specimen.
identified_by	Full name of primary individual responsible for providing the taxonomic identification of the specimen.
taxonomy_notes	General notes regarding the taxonomy of the specimen.
collectors	Comma-separated list of full or abbreviated names of the individuals or teams responsible for collecting the sample in the field.
collection_date_start	The start date for the associated collecting event.
country/ocean	The full, unabbreviated name of the country, major political unit or ocean.
country_iso	Country ISO 3166-1 Alpha-2 code.
region	Park, county, district, lake or river.
site	The name of the sampling location.
coord	Latitude and Longitude in decimal degrees (DD) format (e.g. 45.837).
elev	Elevation of sampling site. Measured in metres relative to sea level. Negative values indicate a position below sea level.
nuc	Nucleotide sequence as submitted.
marker_code	Short code associated with the sequenced marker(gene).
primers_forward	A comma-separated list of forward primer code and sequence pairs, with each code and sequence separated by a colon.
primers_reverse	A comma-separated list of reverse primer code and sequence pairs, with each code and sequence separated by a colon.
sequence_run_site	Full name of the institution where sequencing was performed or the database from which the sequence was retrieved.

## Additional information

This dataset documents the first record of an introduced species in Réunion Island, *Pteris ensiformis* Burm. f. (“Silver lace fern”), which is native to East Asia and Oceania (Holttum 1966). While quite similar morphologically, it is distinguished from other species of the genus by its lamina dimorphism with sword-shaped fertile segments (“ensiform”) with acute and serrated apices and uni-forked veins (Yañez et al. 2023).

With globalisation and fern commercialisation, *Pteris ensiformis* is thought to have been introduced from the 19^th^ century for ornamental uses in the USA (Florida), Mexico, Cuba, Jamaica and Puerto Rico (Lellinger 1985, Martínez 2011, Jones et al. 2018). Considered to have a high naturalisation potential, this species has been recorded with a stable population outside its native range, primarily in South America (Matos et al. 2020). Complementary to these studies, sister species of the *Pteris ensiformis* clade (*Pteris terminalis* Wall. ex J. Agardh and *Pteris insignis* Mett. ex Kuhn) are commonly distributed on other tropical islands, such as the Hawaiian Archipelago and Fiji (Zhang et al. 2014). Consequently, *Pteris ensiformis* can likely establish in diverse tropical habitats and islands.

*Pteris ensiformis* Burm.f was officially observed for the first time on Réunion Island in October 2023 and again in October 2024 in the same location. The observations of the exotic species were situated in Saint-Joseph, Manapany at an elevation of 25 metres and 55 metres, respectively. The location is characterised by ruderals and a high anthropogenic impact area. The specimens were identified, based on morphological characteristics: a) Strongly sterile and fertile frond dimorphism; b) White/grey-green to brown-green lamina, with a pale band across the mid-vein; c) lamina is oblong-ovate and pinnate to bipinnate, with 2–6 pairs of opposite pinnae; d) fertile fronds have pinnae spaced further apart and often 1–3-forked, with narrow, decurrent pinnules that are entire, except at the tips.

The presence of this introduced species was first inferred from morphological identification and subsequently confirmed by molecular data. The rbcL sequence showed a 99.81% match in GenBank to three sequences originating from Taiwan, China and the Philippines, while the trnH–psbA sequence exhibited a 100% match to sequences from French Polynesia, China and Japan.

**Figure 4.**
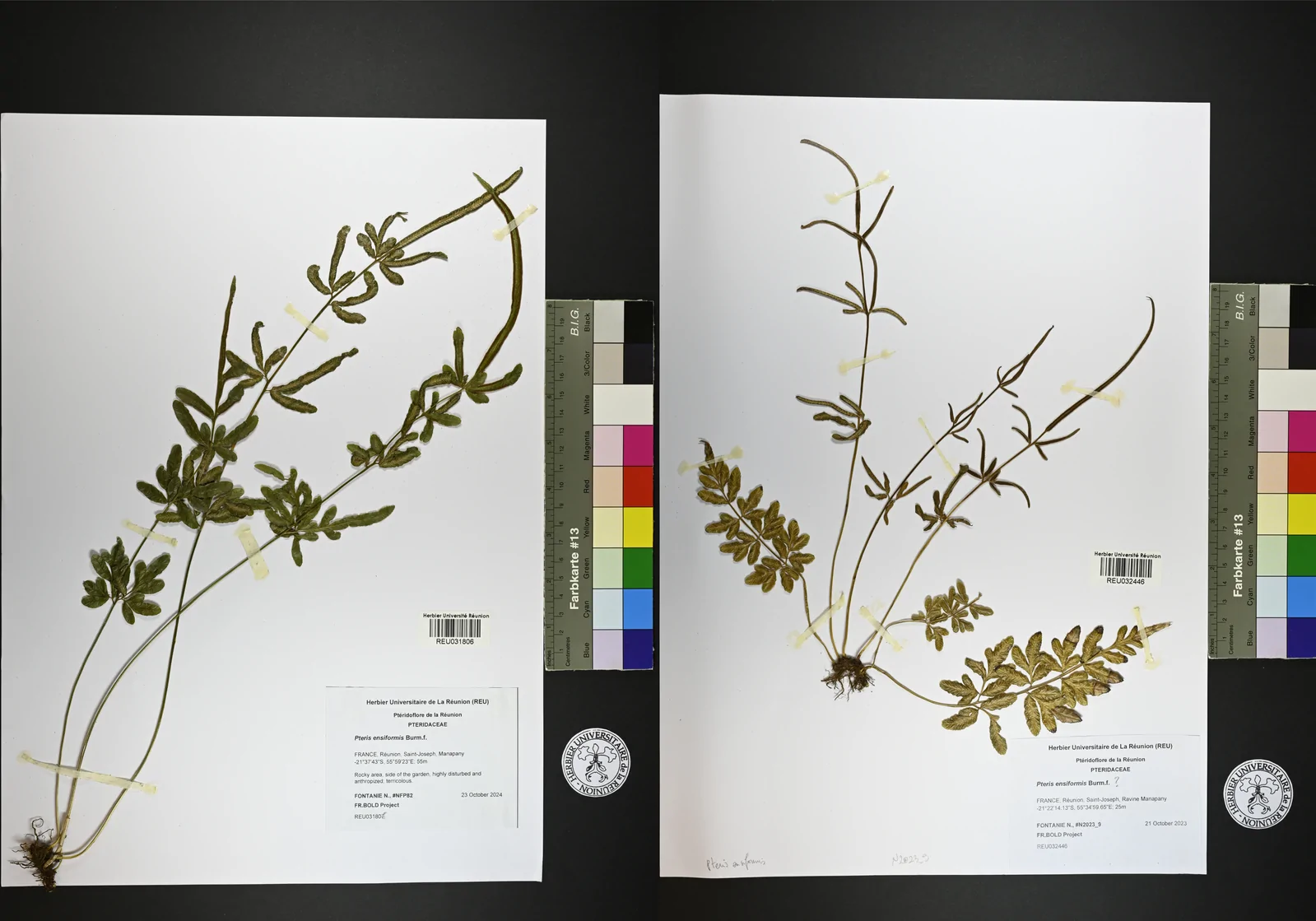
Pictures of specimens of *Pteris ensiformis* Burm.f. (REU031806 and REU032446) stored at REU Herbarium.

Two herbarium sheets accessible at REU herbarium were produced (Fig. 4), showing the species' distinctive traits and would be used to refer to this species' first occurrence in the Indian Ocean Island.

### Conclusions

This dataset constitutes the first publicly available DNA barcode reference library for the pteridophyte flora of Réunion Island. It is based on voucher specimens permanently curated in the REU Herbarium and characterised using the two plastid markers recommended by BOLD for ferns and lycophytes (rbcLa and trnH–psbA). By linking DNA barcode sequences to taxonomically verified herbarium specimens, the dataset provides a robust reference for species identification.

The current version of the dataset was assembled through opportunistic sampling and is, therefore, not intended to provide exhaustive taxonomic or habitat coverage. Several taxa remain unsampled and some habitats are still under-represented. To address these gaps, targeted field surveys are currently being conducted in collaboration with the Conservatoire Botanique National de Mascarin (CBNM), whose long-term knowledge of the distribution of the Island's flora is guiding the selection of indigenous taxa not represented in this first release. The DS-PTEREU dataset will be updated on BOLD as new specimens are collected and sequenced. As the herbarium vouchers and silica gel-preserved tissues are permanently curated at the REU Herbarium and DNA extracts are archived at the CCDB, additional genetic markers can be sequenced from the same material whenever needed to improve species discrimination. As the dataset expands, it will provide a comprehensive resource for taxonomic, biogeographical and conservation studies, including environmental DNA and metabarcoding applications.

## Figures and Tables

**Table 1.** Number of specimens collected per locality and per habitat.

Municipality	Locality notes	Habitat	Number of specimens
Cilaos	Sentier du col du Taïbit	Cliff side	3
		Semi-dry forest	6
		Highland dry forest	8
Plaine des Palmistes	Sentier du piton Cabri	Ridge of a ravine	2
		Seasonal peat land	1
Saint-Benoît	Bélouve - Sentier du Trou de fer	Cloud forest	6
Saint-Joseph	Bord de Rivière Langevin	River side	2
	Grand-Coude, route des Trous	Mid-elevation road ridge	5
		Mid-elevation; disturbed and invaded forest	4
	Grand-Coude, sentier marron	Cloud native forest	13
	Manapany	disturbed and anthropogenised area	6
Saint-Philippe	Mare Longue forest	Lowland moist forest	22
	Pandanaie du littoral	Seashore path	1
	Sentier du tremblet	Lowland moist forest	10
